# Supplementary material for: Identification of photoperiod-induced specific miRNAs in the adrenal glands of Sunite sheep (Ovis aries)
Source: Front Vet Sci. 2022 Jul 22;9:888207. doi: 10.3389/fvets.2022.888207 (PMC9354845; doi:10.3389/fvets.2022.888207)
Supplement: Supplementary file 1 [file Data_Sheet_1.ZIP › Supplementary file/miRDeep2 file.zip!/miRDeep2 file/Know_Structure/SP42A3/oar-mir-3958.pdf]

miRBase precursor : oar-mir-3958
Total read count : 7949
oar-miR-3958-5p read count : 8
oar-miR-3958-3p read count : 7939
remaining reads : 2

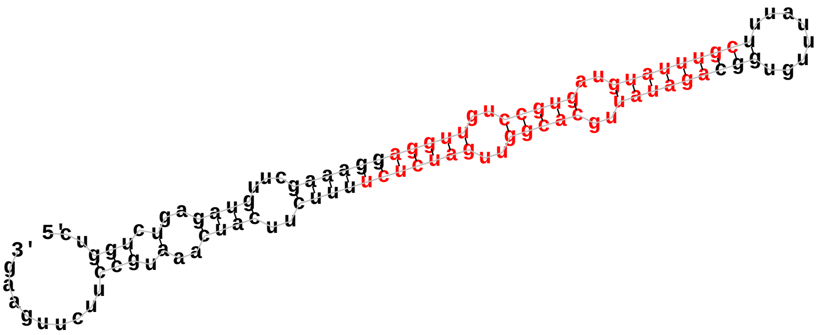

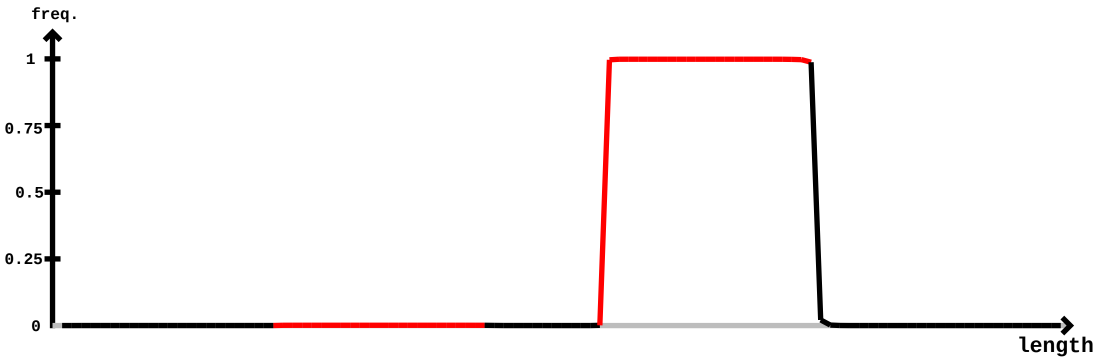


```
                                                 oar-miR-3958-3p
                      oar-miR-3958-5p
5'- cuggucugagauguucgaaaggagguuguccgugauguauuugcuuuauuuguggcagauauugcacgguugaucucuuuucuucaucaaaugccuucuugaag  -3'   exp
    ..(((.(..((((...(((((((((((..(((((..(((((((((........)))))))))..)))))..)))))))))))..))))..).))).........  reads   mm   sample
    .uggucugagauguucgaaagg..................................................................................  1       0    seq
    ......................agguuguccgugauguauuu..............................................................  1       0    seq
    ......................agguuguccgugauguauuugc............................................................  4       0    seq
    ......................agguuguccgugauguauuugcu...........................................................  3       0    seq
    .......................gguuguccgugauguauuugcuuuauu......................................................  1       0    seq
    .......................................................cagauauugcacgguugaucucu..........................  7       0    seq
    ........................................................agauauugcacgguugau..............................  1       0    seq
    ........................................................agauauugcacgguugauc.............................  4       0    seq
    ........................................................agauauugcacgguugaucu............................  8       0    seq
    ........................................................agauauugcacgguugaucuc...........................  77      0    seq
    ........................................................agauauugcacgguugaucucu..........................  7657    0    seq
    ........................................................agauauugcacgguugaucucuu.........................  152     0    seq
    ........................................................agauauugcacgguugaucucuuu........................  13      0    seq
    ........................................................agauauugcacgguugaucucuuuu.......................  2       0    seq
    .........................................................gauauugcacgguugaucucu..........................  17      0    seq
    ..........................................................auauugcacgguugaucucuu.........................  1       0    seq
```
